# Network Modules of the Cross-Species Genotype-Phenotype Map Reflect the Clinical Severity of Human Diseases

**DOI:** 10.1371/journal.pone.0136300

**Published:** 2015-08-24

**Authors:** Seong Kyu Han, Inhae Kim, Jihye Hwang, Sanguk Kim

**Affiliations:** 1 Department of Life Sciences, Pohang University of Science and Technology, Pohang, 790–784, Korea; 2 Department of IT Convergence and Engineering, Pohang University of Science and Technology, Pohang, 790–784, Korea; University of Turin, ITALY

## Abstract

Recent advances in genome sequencing techniques have improved our understanding of the genotype-phenotype relationship between genetic variants and human diseases. However, genetic variations uncovered from patient populations do not provide enough information to understand the mechanisms underlying the progression and clinical severity of human diseases. Moreover, building a high-resolution genotype-phenotype map is difficult due to the diverse genetic backgrounds of the human population. We built a cross-species genotype-phenotype map to explain the clinical severity of human genetic diseases. We developed a data-integrative framework to investigate network modules composed of human diseases mapped with gene essentiality measured from a model organism. Essential and nonessential genes connect diseases of different types which form clusters in the human disease network. In a large patient population study, we found that disease classes enriched with essential genes tended to show a higher mortality rate than disease classes enriched with nonessential genes. Moreover, high disease mortality rates are explained by the multiple comorbid relationships and the high pleiotropy of disease genes found in the essential gene-enriched diseases. Our results reveal that the genotype-phenotype map of a model organism can facilitate the identification of human disease-gene associations and predict human disease progression.

## Introduction

Disease mortality provides important information for the assessment of the clinical severity of a disease within a patient population. Doctors use the mortality information about a specific disease to decide whether to hospitalize patients or improve prevention and early intervention [1]. Diseases with high mortality rates need to be carefully controlled for the overall public health of a country. Policymakers have utilized mortality information to allocate health resources and identify individuals who need urgent health care [2].

The identification of genetic variations associated with clinically severe diseases would allow the prediction of life expectancy. However, we do not have a clear understanding of the genotype-phenotype relationship to predict genetic variations associated with disease mortality. Recent advances in genome sequencing techniques have enabled the identification of genetic variants within an individual. However, the discovery of genetic variations associated with human disease is difficult due to the diverse genetic background of the human population [3,4]. Disease-associated genes detected on the basis of population statistics can explain only a small proportion of disease heritability. Moreover, the detailed molecular mechanisms underlying diseases cannot be studied directly in human subjects due to ethical reasons [3,5,6]. Therefore, the genotype-phenotype map of a model organism could play a complementary role to human population studies, because the genetic background in model organisms can be controlled through the breeding of isogenic lines.

Although model organisms are used frequently for human disease studies, the phenotypic relevance of model organism genes that are orthologous to human disease genes remains unclear [7]. In particular, mutations in an orthologous pair of genes do not always exhibit similar phenotypes in different species [8,9]. For instance, the knockout of *Hprt* exhibits no observable phenotypes in mice, whereas mutations in the human ortholog of *Hprt*, *HPRT1*, cause Lesch-Nyhan syndrome, whose symptoms include the overproduction of uric acid, nervous system impairment, and self-injurious behavior [10].

Modular network analysis of the cross-species genotype-phenotype relationship is helpful to understand the mechanisms underlying human disease progression. It has been suggested that disease phenotypes can be caused by alterations in several genes within a module rather than a single gene mutation [11]. In this concept, a disease module represents a group of disease-associated genes involved in similar phenotypes as well as having molecular connections, such as co-expression, protein interactions, metabolic pathways, co-localizations, and evolutionary constraints [12–14]. Therefore, equivalent phenotypes between different species, called phenologs, might arise from the conservation of functionally related modules that are composed of highly interconnected groups of genes in gene networks [15,16].

Here, we have taken a data-integrative approach to understand the molecular reasons underlying the clinical severity of human diseases. To understand the genotype-phenotype relationship of human diseases, we mapped gene essentiality information from the model organism to human disease genes via their orthology relationship. We discovered that essential and nonessential genes connect diseases in different types, and form clusters in the human disease network. Using a large patient population study, we found that essential gene-enriched disease classes exhibited a higher mortality rate and clinical severity than disease classes enriched with nonessential genes. Moreover, high mortality rate of essential gene-enriched diseases is associated with the high comorbidity of multiple diseases. We found that clinically severe pathological symptoms may be associated with the pleiotropy and high network degree of essential genes in the protein interaction network. Our results suggest that a module-based approach based on the genotype-phenotype map may facilitate the understanding of the progression of human diseases from genetic variation.

## Results

### Relationship between gene essentiality and human diseases

**Fig 1:**
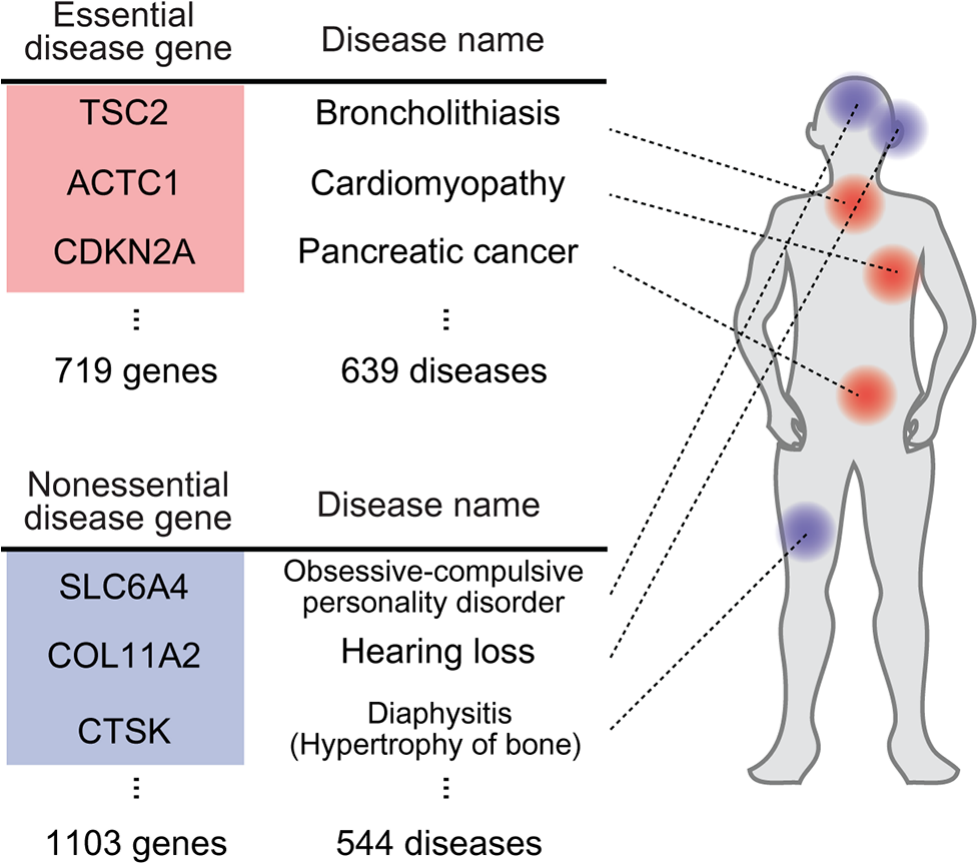
Gene essentiality and human disease genes. Mapping mouse essential and nonessential genes to human disease genes through gene orthologs.

To investigate whether gene essentiality in a model organism is related to human disease phenotypes, we mapped essential mouse genes to human ortholog disease genes (Fig 1; see *Methods*). We selected 2,526 essential genes from mutant gene phenotypes in the Mouse Genome Informatics database (MGI) [17]. We used the mouse as a model organism to define gene essentiality because mutant mouse gene phenotypes are well-annotated from genome-wide analyses of gene deletion models such as knockouts [18]. A gene is considered essential if a mutation of the gene causes a lethal phenotype, which is defined as a developmental failure or a lifespan of less than 50 days [8]. We mapped the essential/nonessential mouse genes to human disease genes in the Online Mendelian Inheritance in Man database (OMIM). Using an ortholog mapping between human and mouse genes, a total of 1,822 disease genes are orthologous with mouse genes. The disease genes are mapped to 713 essential and 1,103 nonessential genes (Fig 1). This procedure enabled us to classify human disease-associated genes that are orthologous to mouse essential and nonessential genes. We categorized these genes as ***essential*** and ***nonessential disease genes***, respectively. These essential and nonessential disease genes are listed in S1 Table.

### Modular organization of the genotype-phenotype map of human disease based on gene essentiality

**Fig 2:**
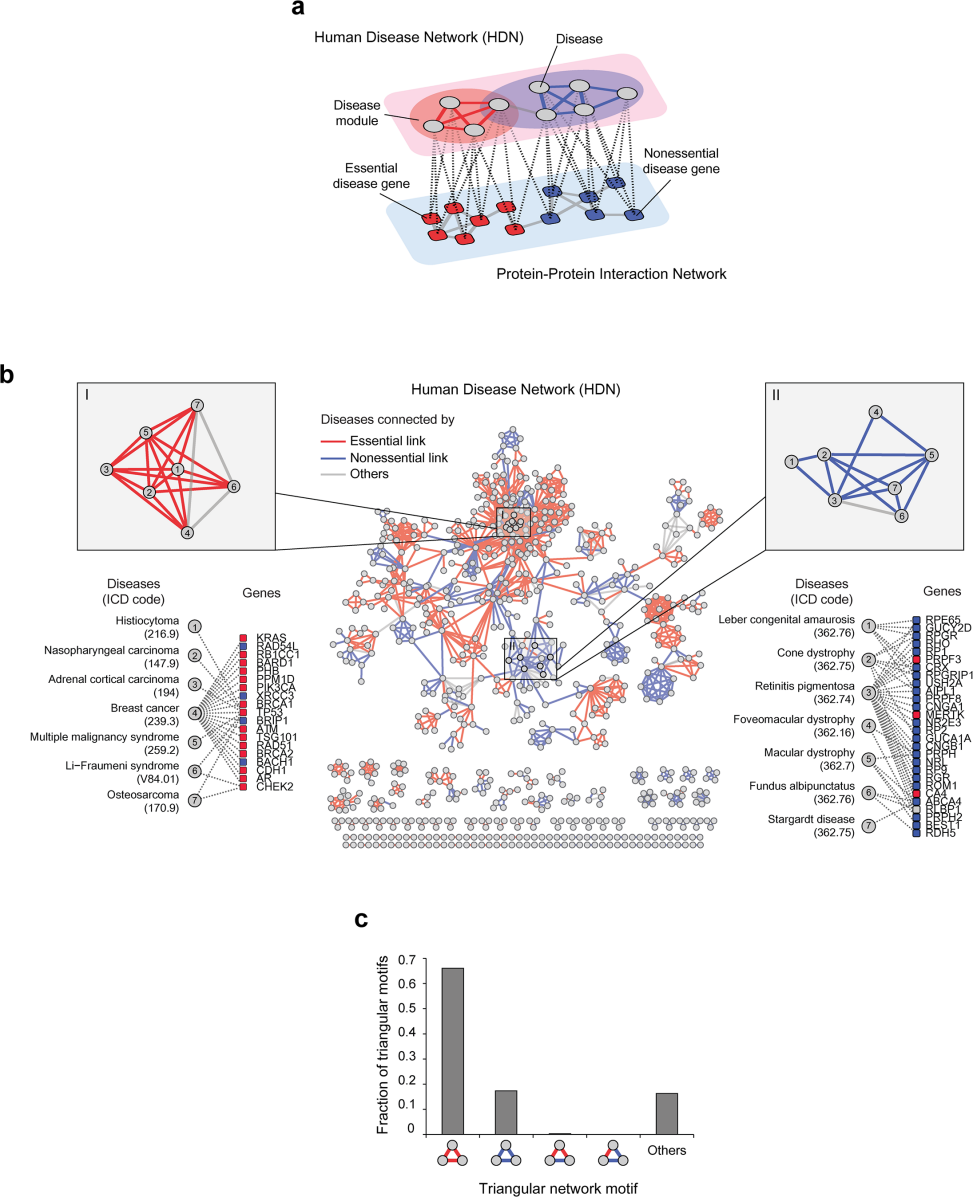
Mapping essential and nonessential disease genes to the Human Disease Network (HDN). (a) The modular architecture of human diseases and their gene essentiality in the HDN. (b) The HDN with the gene essentiality of shared genes is highlighted. Essential/nonessential/other links are colored in red, blue and gray, respectively. Panels I and II show examples of essential and nonessential disease clusters, respectively. (c) The fraction of triangular network motifs connected by essential or nonessential genes. Others are triangular network motifs, where both essential and nonessential disease genes connect minimum two diseases in the network motifs.

We found that essential/nonessential disease genes organize human diseases into a modular structure (Fig 2A). To investigate how the genotype-phenotype relationship organizes disease phenotypes based on gene essentiality, we constructed a human disease network (HDN) linked by essential or nonessential disease genes. HDN nodes represent diseases and links connect them if two diseases have any shared genetic origin [12]. HDN links were classified into essential or nonessential links when the shared genes of disease pair are exclusively essential or nonessential disease genes. HDN links were classified ‘others’ when the shared genes of disease pair include both essential and nonessential disease genes. We supplied the list of essential/nonessential links and shared genes in the HDN (S2 Table).

We found that clusters of diseases are enriched with either essential or nonessential links (Fig 2B). For example, in a disease cluster composed of cancer diseases (panel I), most diseases in the cluster have essential links between them. Indeed, among the genes associated with the disease cluster, 18 of the 23 are essential. Also, the associated genes include *BRCA1*, *BRCA2*, *RAD51* and *TP53*, which are strongly associated with breast cancer [19]. In a disease cluster composed of ophthalmological diseases (panel II), most diseases in the cluster have nonessential links. In this cluster, 24 of the 28 associated-genes are nonessential and include *RP1*, *RPO*, and *RPGR*, which are well-known genes to be associated with sensory perception for light stimuli [20].

Next, we quantified the modularity of the HDN linked by essential/nonessential disease genes. Specifically, we measured the essential and nonessential links in the network motifs. We found that most network triangles were comprised exclusively of either essential or nonessential links (approximately 80% of the total) (Fig 2C). The triangular network motif is a basic component of network clusters and the smallest network motif that comprises a complete sub-graph [21]. To validate if the pattern of network motifs is affected by gene essentiality, we compared the fraction of network motifs with random control. Gene essentiality information is randomly shuffled across disease genes. We confirmed that the observed fractions of network motifs connected by essential links are indeed higher than expected (S1 Fig).

### Gene essentiality and the clinical severity of disease classes

**Fig 3:**
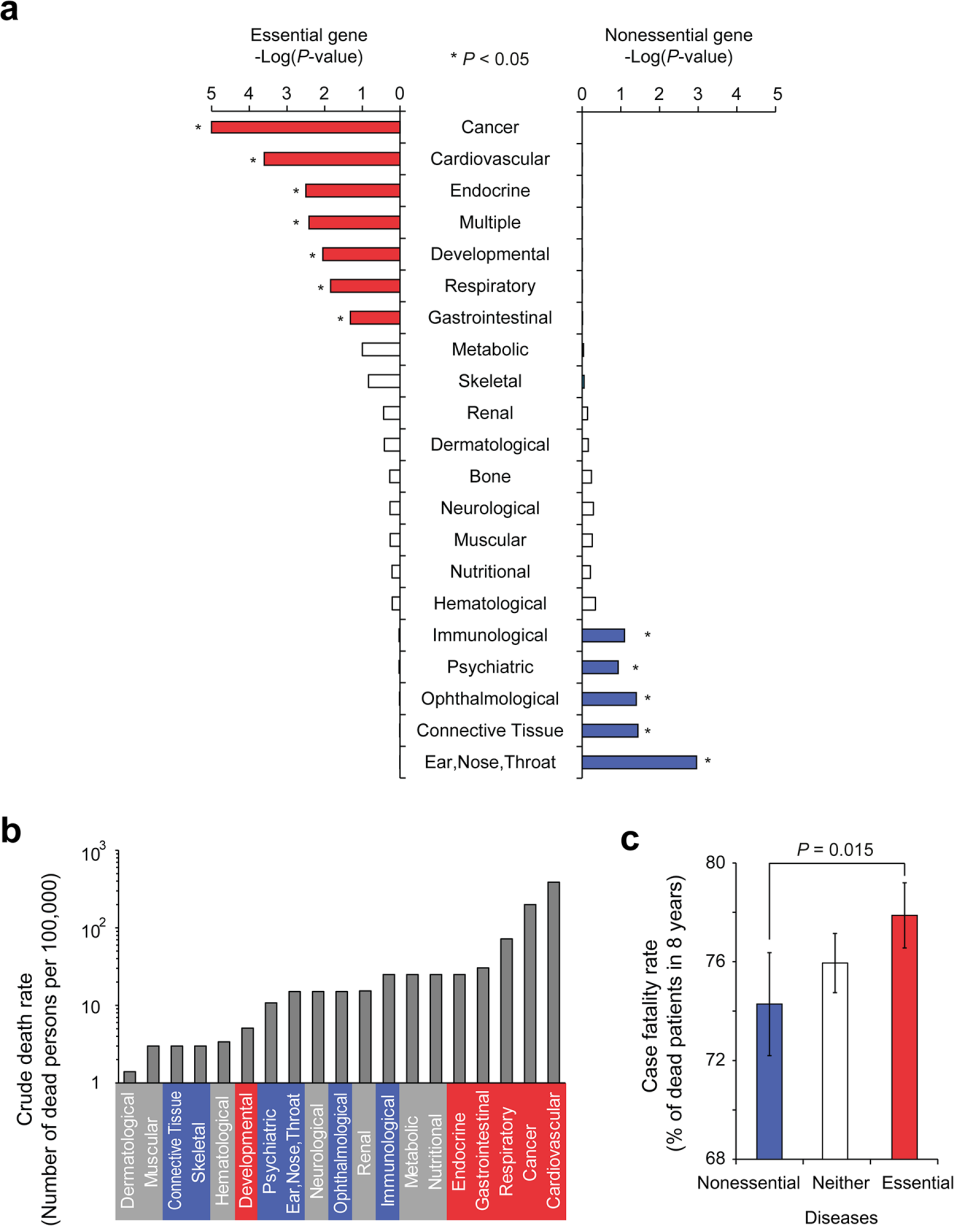
The mortality of human disease classes according to gene essentiality. (a) Human disease classes sorted by gene essentiality. The enrichment of essential and nonessential genes in 21 human disease classes is shown. * indicates *P* < 0.05 (Hypergeometric *P*-values). (b) The crude death rate of different disease classes. The crude death rate is calculated as the number of deaths reported each calendar year per 100,000 individuals. Disease classes that are enriched with essential and nonessential genes are colored in red and blue, respectively. (c) The case fatality rate of diseases differentially enriched with essential and nonessential genes. The case fatality rates of diseases enriched with neither essential nor nonessential genes are also shown as “Neither”.

Gene essentiality configures the genotype-phenotype map of human diseases in a modular manner, as shown in Fig 2. Thus, we transferred gene essentiality onto the disease classes, because disease classes are clustered in human disease network and grouped in terms of phenotypic similarity based on the affected physiological system [12]. We discovered that disease classes are biased toward the enrichment of essential or nonessential genes. Specifically, genes in different disease classes are differentially enriched in either essential or nonessential disease genes (Fig 3A). Genes in cancer, cardiovascular, endocrine, developmental, respiratory, and gastrointestinal disease classes as well as diseases that involve multiple systems are enriched with essential disease genes (*P* < 0.05 in the hypergeometric distribution). In contrast, genes in ear-nose-throat, connective tissue, ophthalmological, psychiatric, and immunological disease classes are enriched with nonessential disease genes (*P* < 0.05 in the hypergeometric distribution).

We investigated disease mortality in patients who carried diseases from disease classes enriched with essential or nonessential disease genes (see *Methods*). We found that diseases enriched with essential genes were associated with a higher crude death rate than diseases enriched with nonessential genes (Fig 3B). The crude death rate for a specific disease was based on an analysis of the number of deaths per 100,000 individuals [22]. Among the six disease classes that were identified to be enriched with essential genes, five disease classes (cardiovascular, cancer, respiratory, gastrointestinal, and endocrine) were associated with a high crude death rate. Developmental diseases, which were also identified to be enriched with essential genes, were associated with low crude death rates. However, only live births were included in the calculation of death rates, which introduces a selection bias because many cases that could have led to death may have been aborted at the fetal stage [22]. The total crude death rates due to developmental diseases may therefore be much higher than current studies suggest.

We also found that diseases enriched with essential genes have higher case fatality rates than diseases enriched with nonessential genes. Case fatality rate is the proportion of patients that die from a particular disease within a specified period of time (see *Methods*). For example, patients with essential gene-enriched diseases were more likely to be deceased within 8 years of the initial diagnosis (Fig 3C). According to Medicare records, on average 76% of patients were deceased within 8 years for all disease types. However, essential gene-enriched diseases were associated with a higher clinical severity than nonessential gene-enriched diseases (*P* = 0.015, Mann-Whitney *U* Test). The list of essential and nonessential gene-enriched diseases and their clinical severity is provided in S3 Table. These results suggest that the gene essentiality map of a model organism can be used to predict the clinical severity of human diseases.

We found that the diseases enriched with essential genes have higher case fatality rates (Fig 3C). Among them we discovered cancers and cardiovascular diseases, which are often manifested in elderly patient group. Because we used medical claims associated with elderly patients (≥ age 65), one might ask whether the fatality rate data could have a bias toward disease classes frequently found in elderly patients. Therefore, we tested the potential bias in the dataset by counting the number of the patients from the disease classes enriched in essential/nonessential genes. We confirmed that the number of patients do not have bias towards diseases associated with essential or nonessential genes (S2 Fig; *P* = 0.23, Mann-Whitney *U* Test). Furthermore, the conclusion is reconfirmed by the crude death rate which comes from a different data source (Fig 3A). Thus, we believe that the association between disease fatality and gene essentiality to be true, although the case fatality data should be interpreted with care.

### Essential gene-enriched diseases are associated with high comorbidity and pleiotropy

Why do essential genes tend to associate with mortal diseases? The high disease mortality in essential gene-enriched diseases also may be due to comorbid diseases. Disease comorbidity is the co-occurrence of other diseases with a primary disease. It has been shown that the patients affected by disease having many comorbid diseases tended to die sooner [23].

**Fig 4:**
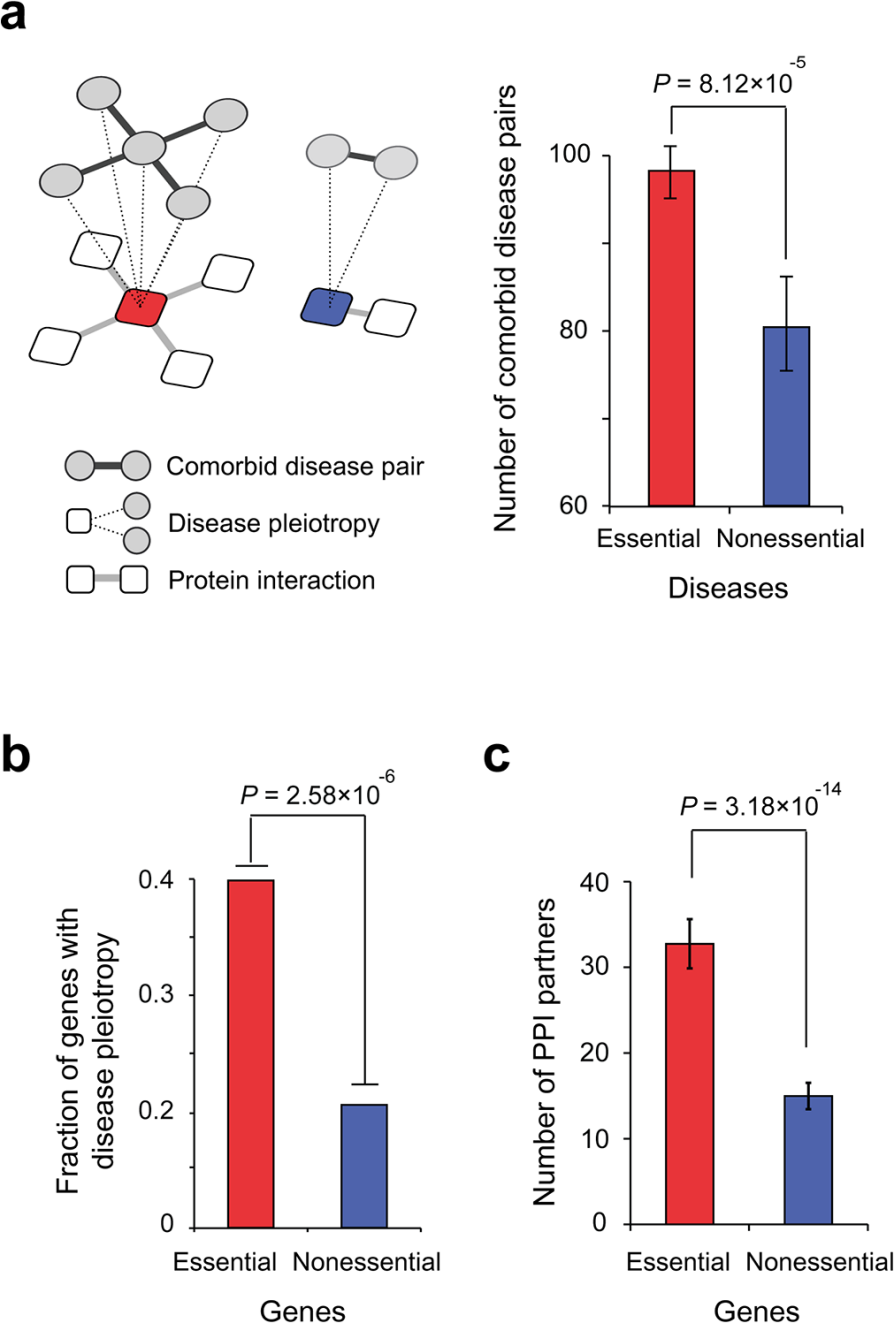
The comorbidity and pleiotropy of essential and nonessential gene-enriched diseases. (a) The comorbid disease pairs of essential and nonessential gene-enriched diseases. (b) The fraction of pleiotropic diseases connected by essential and nonessential genes. (c) The number of PPI partners of essential and nonessential disease genes.

We therefore investigated the relationship between comorbidity and gene essentiality. We found that diseases enriched with essential genes have a higher number of comorbid disease pairs compared to diseases enriched with nonessential genes (Fig 4A; *P* = 8.12×10^−5^, Mann-Whitney *U* test). To quantify the number of comorbid disease pairs, we counted the number of diseases that co-occurred with a particular disease compared to random expectation in a patient population (see *Methods*). This result suggests that diseases caused by mutations in essential genes are more likely to progress to clinically severe conditions with multiple comorbid diseases.

Next, we analyzed disease pleiotropy in human genetic diseases as another potential cause of multiple pathological symptoms. Genes with disease pleiotropy are associated with two or more genetic disorders and may cause multiple pathological defects due to the involvement of more diverse cellular functions [24]. We quantified the genes that exhibited disease pleiotropy and found that essential genes tend to exhibit disease pleiotropy more frequently than nonessential genes (Fig 4B; *P* = 2.58×10^−6^, Fisher’s exact test). High comorbidity and disease pleiotropy suggest that essential genes connect a larger number of disease phenotypes via molecular connections than nonessential genes.

If a gene has more connections in the cellular network (*i*.*e*., the gene is a hub gene), then its perturbation tends to result in the disconnection of multiple cellular function, which leads to disease pleiotropy. Jeong et al. [25] previously reported that essential genes in another model organism, *Saccharomyces cerevisiae* (yeast), have more PPI partners than nonessential genes. We expanded this analysis to examine the PPIs of human disease genes, and found that essential disease genes have more PPI partners than nonessential disease genes (Fig 4C; *P* = 3.18×10^−14^, Mann-Whitney *U* Test). The higher pleiotropy and connectivity of essential disease genes supports the observation that perturbations of these genes cause more lethal effects in the cellular network and explains the clinical severity of essential gene-enriched diseases.

## Discussion

We found that gene essentiality derived from the mouse model organism is correlated with the clinical severity of human diseases despite the evolutionary distance between mice and humans. Our results suggest that a module-based approach using genotype-phenotype mapping of a model organism may provide a better understanding of the genetic variations that lead to human diseases. Specifically, human disease classes tend to be more clinically severe if their associated genes are enriched in essential genes (Figs 2 and 3).

A modular architecture was found in the genotype-phenotype map of human diseases along with gene essentiality (Fig 2). Although we utilized gene-to-gene orthology to map the mouse essential genes to human disease genes, the observed modular architecture of human diseases might be rooted in a conservation of modules in the genotype-phenotype map in evolution. The modules in the genotype-phenotype map are known to have emerged during the course of evolution [26,27] because the modular architecture may reduce the potential deleterious effects of mutations, which otherwise may spread and threaten the survival of the organism [27,28].

We found that nonessential gene-enriched diseases were less clinically severe than essential gene-enriched diseases (Fig 3). This result indicates that nonessential gene-enriched diseases may affect quality of life rather than mortality. This observation implies that it may be necessary to systematically screen phenotypes in the model organism in adulthood to better understand disease phenotypes. For example, the serotonin neurotransmitter transporter *SLC6A4* is a mouse nonessential gene, and mutations to the human ortholog of this gene cause obsessive-compulsive disorder through a defect in the regulation of serotonin levels [29]. Only an extensive screening of behavior enabled the detection of a phenotype in which the *SLC6A4* knockout mouse exhibited obsessive behavior, including continuous and progressive grooming. A mutation in another mouse nonessential gene, *FAM107B*, also was recently reported to display a deafness phenotype. Extensive screening of the signal to the brain in response to auditory stimulation was necessary to detect such a phenotype [30]. Currently, several mouse phenotypes are being systematically screened, and diverse phenotypes in the adult stage of model organisms have been identified [31–33]. Therefore, we anticipate that our approach can be expanded toward understanding diseases that affect quality of life.

## Methods

### Essential and nonessential gene data set

Essential and nonessential genes were compiled from the mutant phenotypes listed in the Mouse Genome Informatics database (www.informatics.jax.org, downloaded in 2012) [17]. These mutant phenotypes were documented from knockout, trapping, or random mutagenesis studies. Genes were classified as essential genes if the mutant phenotype exhibited a severe effect, namely "embryonic lethality" (MP: 0002080), "prenatal lethality" (MP: 0002081), "postnatal survival lethality" (MP: 0002082), "abnormal reproductive system morphology" (MP: 0002160), or "abnormal reproductive system physiology" (MP: 0001919). The remaining genes in the mouse genome were classified as nonessential genes. A total of 2,526 essential and 15,014 nonessential genes were identified from the mouse.

### Comparative genomic analysis between humans and mice

The orthologous relationship between human and mouse genes was predicted by the sequence homology. The human and mouse genomes were curated from the National Center for Biotechnology Information NCBI36 and NCBIM36 databases. The mouse orthologs, including sequences, of the human genes were extracted using EnsemblCompara GeneTrees [34], which is downloaded in 2012 via Biomart (http://www.biomart.org). Consequently, one-to-one orthologs of 14,223 genes were detected between humans and mice.

### Human disease genes and phenotypes

Human gene and disease phenotype associations were curated from the OMIM database (http://www.ncbi.nlm.nih.gov/omim/, 2009 version) [35]. The OMIM database provided gene-disease associations between the 2,929 disease types in the Morbid Map and 1,777 disease-associated genes. According to Goh et al [12], disease subtypes were combined into a single disease based on a string match of disease annotations because some disease types have minor differences in their names. A total of 1,228 unique diseases were extracted from 2,161 disease annotations [12]. Human diseases were classified by Goh et al [12] into 21 different disease classes based on the physiological systems associated with each disease. The disease classification is based on the phenotype level because this type of classification has been shown to discriminate phenotypic similarities and differences based on disease symptoms [36].

### Mortality rate from human patient population studies

The case fatality rate of a disease was measured as the percentage of patients who were deceased within 8 years from the initial diagnosis of the disease. According to Hidalgo et al [23], the disease progression data were compiled from 13,039,018 patients in MedPAR, a database that includes elderly Americans aged 65 or older who were enrolled in the Medicare program from 1990 to 1993. The crude death rate was measured as the number of patients deceased from that disease per 100,000 individuals in the U.S. population. The crude death rate data were extracted from the compressed mortality data from 1979 to 1998 with ICD-9 codes (http://wonder.cdc.gov/cmf-icd9.html) reported by the U.S. Centers for Disease Control and Prevention (CDC) [22].

### Identification of comorbid disease pairs

The number of comorbid disease pairs was quantified by relative risk (*RR*). If a disease pair had an *RR* ≥ 2, then the pair was counted as a comorbid disease pair. RR ≥ 2 was known as a baseline of comorbidity value whose disease pairs are linked in the HDN [37]. The *RR* quantifies the co-occurrence of two diseases compared to random expectation in the human patient population. The *RR* of diseases *i* and *j* is given by:

$$RR=\frac{C_{ij}}{C^{*}{}_{ij}}$$ (1)

where *C*_*ij*_ is the number of patients who had both disease *i* and disease *j*, and *C*_ij_* is equal to *I*_*i*_ *I*_*j*_ */N*, which represents random expectation. *I*_*i*_ is the incidence of disease *i*. *N* is the total number of patients (13,039,018) in the Medicare record.

### Construction of the PPI network

We compiled human protein interactions from a total of 22 existing protein interaction databases: the Bio-molecular Interaction Network Database (BIND) [38], the Human Protein Reference Database (HPRD) [39], the Molecular Interaction database (MINT) [40], the Database of Interacting Proteins (DIP) [41], IntAct [42], BioGRID [43], Reactome [44], the Protein-Protein Interaction Database (PPID), BioVerse [45], CCS-HI1 [46], the Comprehensive Resource of Mammalian protein complexes (CORUM) [47], IntNetDB [48], the Mammalian Protein-Protein Interaction Database (MIPS) [49], the Online Predicted Human Interaction Database (OPHID) [50], Ottowa [51], PC/Ataxia [52], Sager [53], Transcriptome Complex [54], Unilever, a protein-protein interaction database for PDZ domains (PDZBase), and a protein interaction data set from the literature [55]. We removed low-confidence interactions that were not supported by direct experimental evidence. The final network included 101777 interactions between 11,043 human proteins. We supplied the integrated PPI network (S4 Table).

## Supporting Information

S1 Fig The distribution of triangular network motifs connected by (a) all essential link, (b) nonessential link, (c, d) both essential and nonessential link and (e) others with random control. (TIF)

S2 Fig Comparison of the number of patients of (a) diseases associated with essential or nonessential genes (b) diseases class enriched with essential, nonessential or neither gene. (TIF)

S1 Table The list of essential and nonessential disease genes. (XLS)

S2 Table The list of essential/nonessential links and shared genes in HDN. (XLS)

S3 Table The case fatality rate of human diseases enriched with essential and nonessential genes. (XLS)

S4 Table The list of protein interactions from integration of 22 protein interaction databases. (TXT)
