# Supplementary figures and images for: Network Modules of the Cross-Species Genotype-Phenotype Map Reflect the Clinical Severity of Human Diseases
Source: PLoS One. 2015 Aug 24;10(8):e0136300. doi: 10.1371/journal.pone.0136300 (PMC4547739; doi:10.1371/journal.pone.0136300)

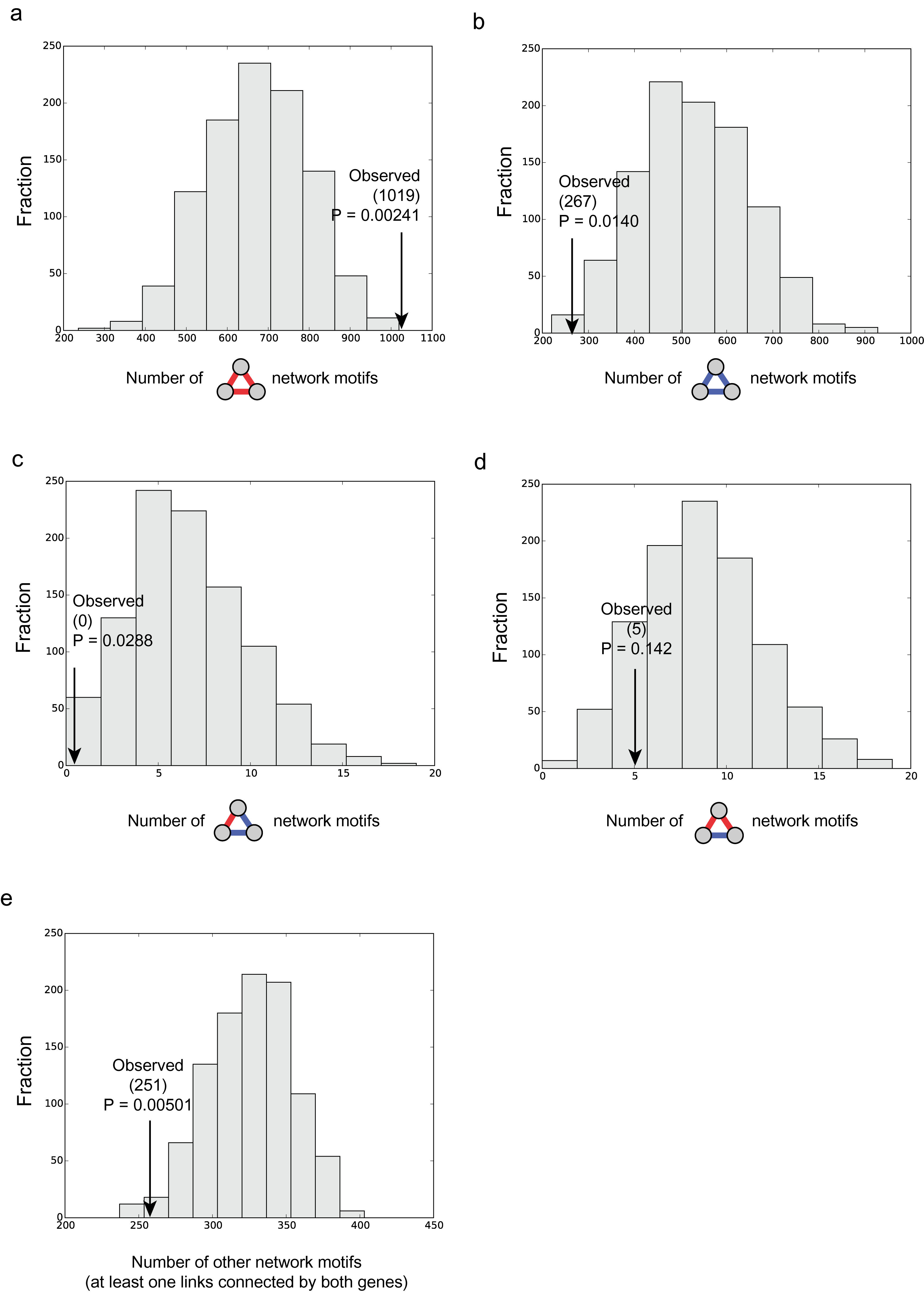

Supplement: S1 Fig — (TIF) [file pone.0136300.s001.tif]

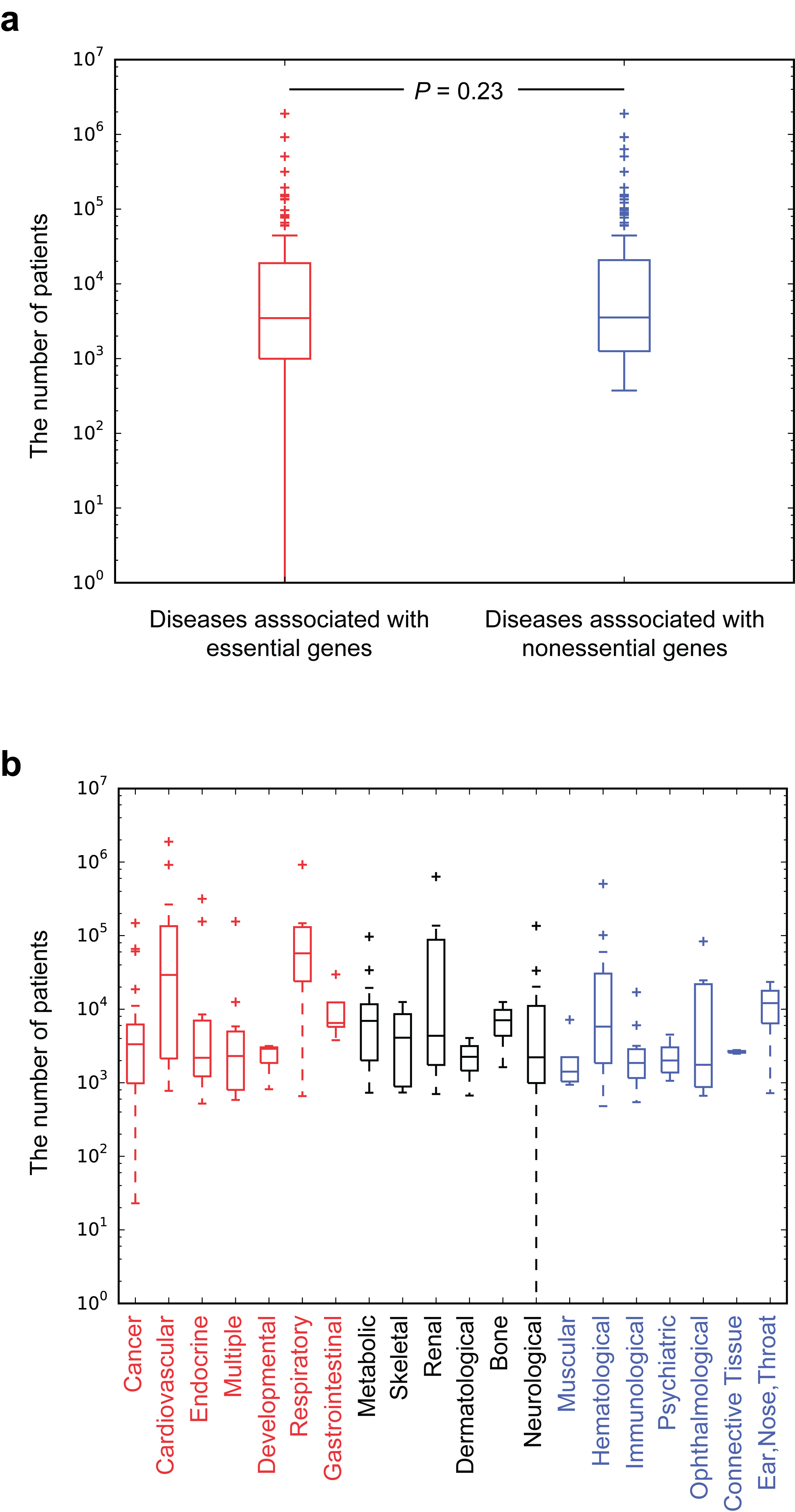

Supplement: S2 Fig — (TIF) [file pone.0136300.s002.tif]
